# Synthesis and Physicochemical Properties of Thermally Sensitive Polymeric Derivatives of *N*-vinylcaprolactam

**DOI:** 10.3390/polym16131917

**Published:** 2024-07-05

**Authors:** Agnieszka Gola, Rafał Pietrańczyk, Witold Musiał

**Affiliations:** Department of Physical Chemistry and Biophysics, Pharmaceutical Faculty, Wroclaw Medical University, Borowska 211, 50-556 Wroclaw, Poland; agnieszka.gola@umw.edu.pl (A.G.); rafalpietranczyk17@wp.pl (R.P.)

**Keywords:** *N-vinylcaprolactam*, lower critical solution temperature, anionic initiator, potassium persulfate, electrical conductivity, dynamic light scattering

## Abstract

Six derivatives of poly-*N*-vinylcaprolactam (PNVCL) P1-P6 were synthesized via surfactant-free precipitation polymerization (SFPP) at 70 °C, with potassium persulfate (KPS) as the initiator. P5 and P6 were synthesized using the cross-linker *N*,*N*′-Methylenebisacrylamide (MBA). The conductivity was measured to monitor the polymerization process. The hydrodynamic diameters (HDs) and polydispersity indexes (PDIs) of aqueous dispersions of P1-P6 were determined using dynamic light scattering (DLS) and zeta potential (ZP) using electrophoretic mobilities. At 18 °C for P1–P6, the HDs (nm) were 428.32 ± 81.30 and PDI 0.31 ± 0.19, 528.60 ± 84.70 (PDI 0.42 ± 0,04), 425.96 ± 115.42 (PDI 0.56 ± 0.08), 440.34 ± 106.40 (PDI 0.52 ± 0.09), 198.39 ± 225.35 (PDI 0.40 ± 0.19), and 1201.52 ± 1318.05 (PDI 0.71 ± 0.30), the and ZPs were (mV) 0.90 ± 3.23, −4.46 ± 1.22, −6.44 ± 1.82, 0.22 ± 0.48, 0.18 ± 0.79, and −0.02 ± 0.39 for P1–P6, respectively. The lower critical solution temperature ranged from 27 to 29 °C. The polymers were characterized using the ATR-FTIR method. The study concluded that the physicochemical properties of the product were significantly affected by the initial reaction parameters. Polymers P1-P4 and P6 have potential for use as drug carriers for skin applications.

## 1. Introduction

Stimulus-sensitive polymers are polymers that undergo fully controlled, predictable, and reversible physical or chemical transformations in response to small external changes in environmental conditions [1,2,3,4,5,6,7]. Chemical stimuli can affect interactions between polymer chains or solvents at the molecular level. This occurs when the concentration of hydrogen ions or ionic agents changes. Physical stimuli affect the level of various energy sources and alter molecular interactions at critical starting points. These stimuli can include temperature, electric or magnetic fields, and mechanical stresses [8,9,10,11,12,13].

Temperature is a crucial physiological factor in the body. Some diseases are manifested by a change in temperature. Therefore, temperature-responsive polymers have been of great interest among all the stimulus-responsive polymers studied [14,15]. Thermosensitive polymers exhibit a decrease in solubility in water when the temperature rises above the lower critical solution temperature (LCST) and does not exceed the upper critical solution temperature (UCST) [16,17,18,19,20]. At the LCST, the polymer undergoes a transition from hydrophilic to hydrophobic equilibrium, resulting in reduced solubility in water. The phase transition temperature depends, i.a., on the molar mass of the polymer molecule and other components that make up the polymer chain [21]. This temperature may be altered and controlled by changing the amount of initiator, incorporating copolymers in the polymer chain, or using a cross-linking agent [22,23,24]. Incorporation of hydrophilic co-monomers may favor the increase of phase transition temperature. The increase is driven by hydrophilic interactions, which require respective levels of thermal energy to destruct the solvation sphere. Conversely, reduction in the number of hydrophilic co-monomers, while increasing hydrophobicity, can lower the phase transition temperature [25,26,27,28]. Temperature-sensitive polymers have enabled significant progress in tissue engineering and drug delivery [29,30,31,32]. As drug carriers, they offer improved properties, including better control over drug release rates and reduced toxicity, compared to traditional drug forms. These properties lead to better therapeutic outcomes [33,34,35,36].

Poly *N*-vinylcaprolactam (PNVCL) is one of the most popular thermosensitive polymers for use as an experimental drug carrier, as well as poly *N*-isopropylacrylamide (PNIPA) [37]. Both of these polymers are considered smart polymers due to their fully reversible and controlled temperature sensitivity [38]. It was initially presented that the phase transition temperature of PNVCL coincided with that of PNIPA (32–34 °C). However, the range of phase transition temperatures for PNVCL is much wider, typically between 30 and 50 °C, and is strongly dependent on the polymer’s molecular weight and concentration [39,40,41,42,43]. Currently, PNVCL is considered biocompatible, indicating that it has no negative impact on the body and does not result in toxicity, hemolysis, or immune system disruption. Polymers with these characteristics have made significant progress in the field of biomedicine, offering a range of diagnostic and therapeutic possibilities [44,45,46,47,48]. PNVCL exhibits excellent biofilm-forming properties and can complex organic molecules, making it a suitable carrier for various chemicals, particularly drugs [49].

Treating diseases, including cancer, is a challenging task that requires a constant search for alternative treatments and new chemical entities. The research to discover and bring drugs to market is a lengthy and expensive process. Therefore, the pharmaceutical industry is exploring ways to better utilize the potential of known compounds. One such approach is to use smart polymers as drug carriers. Thermosensitive polymers are a suitable choice for carrier materials due to their ability to undergo a phase transition at a specific temperature. The PNVCL polymer is a promising drug carrier for targeted therapy because of its thermosensitivity, biocompatibility, and biodegradability, which can improve pharmacokinetic parameters.

The aim of this study was to assess the effect of anionic initiator concentration and co-monomer presence on the course PNVCL polymerization and its physicochemical properties. The effects of temperature on the hydrodynamic diameter, polydispersity index, and zeta potential of the polymer solutions were examined. The pH values of the solutions were also measured, and infrared spectroscopy studies were performed.

The measurements obtained from these investigations serve to provide a fundamental characterization of the resulting particles and to generate valuable scientific data that can be utilized in various fields of polymer chemistry [50,51,52,53,54].

## 2. Materials and Methods

### 2.1. Materials

*N*-vinylcaprolactam (NVCL, 98%, St. Louis, MO, USA), potassium persulfate (KPS, 98%, Sternheim, Germany), *N*,*N*′-Methylenebisacrylamide (MBA, 97%, St. Louis, MO, USA), and dialysis tubing cellulose membrane (MWCO 12,000–14,000 Da St. Louis, MO, USA) were obtained from Sigma Aldrich. The water used in this experiment was deionized (<0.06 μS cm^−1^) and filtered through an HLP 20 system (microfiltration capsule 0.22 μm, Hydrolab, Straszyn, Poland) to meet the requirements of the PN-EN ISO 3696:1999 [55] standards for analytical laboratories. The chemicals were used as received without any further purification or modification.

### 2.2. Synthesis

Six derivatives of thermosensitive polymeric *N*-vinylcaprolactam namely P1, P2, P3, P4, P5, and P6 were synthesized using the surfactant-free precipitation polymerization method originally developed by Pelton [56]. The polymerization was conducted in deionized water using potassium persulfate (KPS) as the anionic initiator and the methylene bisacrylamide (MBA) as the cross-linking agent. The reaction was carried out under a nitrogen atmosphere at 70 °C for 6 h in a total volume of 1000 mL. The experiment was performed in a 2000 mL round bottom flask with four necks. The flask was equipped with a 300 mm Allihn condenser, a nitrogen inlet and outlet, a temperature sensor, a conductivity cell with K = 1 cm^−1^, and a magnetic stirring bar. The flask was heated in a water bath while stirring at 250 rpm. The required initiator sample was added to a reaction vessel containing 900 mL of deionized water at 70 °C. The mixture was continuously stirred and degassed with nitrogen bubbles for about 10 min. Then, the monomer (dissolved in 50 or 100 mL of water) and the co-monomer (dissolved in 50 mL of water) were added to the reaction vessel, initiating the polymerization reaction. Table 1 lists the reaction conditions, including acronyms for the substrates.

Each reaction mixture (170 mL) was purified by forced equilibrium dialysis (FED) against 2000 mL of freshly deionized mixed water for approximately six days, with daily water changes, in semipermeable cellulose membrane tubes (MW cut-off 10–12 kDa, 43 mm diameter). The water’s conductivity was measured before each water change. The purification process was considered complete when the conductivity measurement was around 1.3–1.6 µS cm^−1^ for two consecutive water exchange cycles. After purification, the samples underwent pH, HD, and ZP testing. They were then stored in dark glass bottles at room temperature for future use.

Each purified polymer suspension, approximately 100 mL in volume, was placed in sample containers, frozen, and then freeze-dried in an Alpha 1-2 LD (Martin Christ Freeze Dryers, Osterode am Harz, Germany) for 26 h. The resulting dry copolymer products were characterized using the ATR-FTIR technique.

The morphology of the resulting precipitate in the P5 synthesis was observed under an optical microscope called Stereo Zoom Microscope SMZ-171-TLED (Motic, Hong Kong, China) at fifty times magnification.

**Figure 1:**
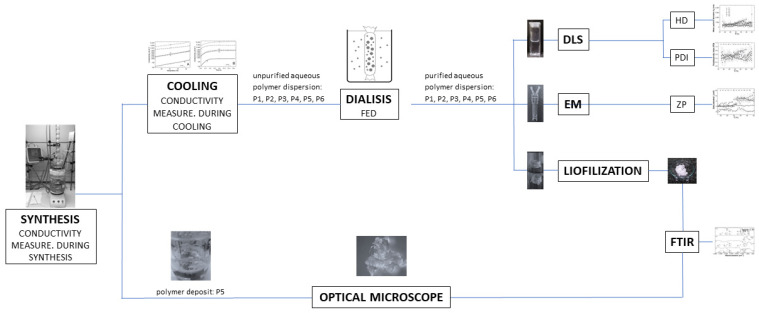
The general scheme of the experimental studies. FED—forced equilibrium dialysis, DLS—dynamic light scattering, EM—electrophoretic mobility, HD—hydrodynamic diameter, PDI—polydispersity index, ZP—zeta potential, FTIR—Fourier transform infrared spectroscopy.

The experimental part of the project is shown as a flow chart in Figure 1.

### 2.3. Conductivity Measurements

The conductivity of the reaction mixture was measured using a CC-505 conductivity meter (Elmetron, Gliwice, Poland) with an accuracy of up to 19,999 mS cm^−^^1^ ± 0.1% and from 20,000 mS cm^−^^1^ ± 0.25%. The measurements were taken during the polymerization reaction at a constant temperature of 70 °C and as a function of temperature during the cooling process. The conductivity meter used a platinum electrode, a glass housing EC-60 immersion conductivity sensor (K = 1.0 ± 0.2 cm^−^^1^, Elmetron, Gliwice, Poland), and a Pt-1000A temperature sensor (0–100 ± 0.35 °C). Both sensors were continuously immersed in the reaction mixture. Temperature compensation was provided manually during the polymerization reaction and automatically during cooling.

### 2.4. pH Measurements

The pH of six synthesis products was investigated, including dispersions of unpurified and purified polymers. The pH was measured at room temperature using an ELMETRON CPC-511 pH meter (pH range: −2.00 to 16.00, accuracy: ±0.01 pH, Elmetron, Gliwice, Poland) equipped with an ELMETRON EPS-1 electrode. The samples were not diluted or buffered.

### 2.5. Attenuated Total Reflection Fourier-Transformed Infrared Spectroscopy Measurements (ATR-FTIR)

The Nicolet iS50 FT-IR spectrometer, equipped with a universal ATR sampling accessory composed of monolithic diamond crystals (Thermo Fisher Scientific, Madison, WI, USA), was used to perform Attenuated Total Reflection Fourier Transform Infrared Spectroscopy (ATR-FTIR). The radiation was recorded with the L-alanine-doped deuterated triglycene sulphate detector (DLaTGS) at a wave number resolution of 4 cm^−^^1^ ± 0.01 cm^−^^1^. The ATR-FTIR spectra were obtained by averaging 32 scans per sample cycle in the wavelength range of 4000 to 400 cm^−^^1^. The background spectra were automatically subtracted, and reference spectra were recorded using a blank ATR crystal after cleaning the ATR module and before sample application. The ATR element and pressure clamp were washed with methanol and dried multiple times. The ATR-FTIR spectra of the substrates in their commercial form and the lyophilized polymerization products were measured at ambient temperature. A small amount of the solid sample was placed directly on the flat surface of a monolithic diamond crystal cell and pressed down using a clamp with manual adjustment of the total compressive force applied to the sample. The measurements were carried out under the same conditions. The ATR-FTIR spectral data were analyzed using the OMNIC software (version 9, Thermo Fisher Scientific, Madison, WI, USA).

### 2.6. Hydrodynamic Diameter (HD) and Polydispersity Index (PDI) Measurements

The Zetasizer Nano ZS ZEN3600 device (Malvern Instruments, Malvern, UK) equipped with the standard red He-Ne laser (4 mW, λ = 633 nm) was used to measure the hydrodynamic diameter (HD), distributions, and polydispersity index (PDI) of the aqueous polymer particle dispersion by the dynamic light scattering (DLS) method. Light scattering measurements were taken using a sensitive avalanche photodiode detector (APD) positioned at a 173° angle with non-invasive backscattering (NIBS) technology. The light intensity during the measurement was regulated by automatically adjusting the laser beam attenuation. The measurements were taken in an optically translucent polyacrylic disposable DTS-0012 cuvette (Malvern Instruments, Malvern, UK) filled with 1 mL of the sample purified by dialysis without precipitation and not diluted. The cuvette was placed in the temperature-controlled measurement cell and equilibrated for 240 **s** before taking measurements at each new temperature. The DLS measurements were recorded from 18 to 45 °C in 1 °C increments. The number of runs per measurement was automatically adjusted to a range of 10–100. The measurement runs were automatically adjusted to between 10 and 100. The HD and PDI values were estimated using the cumulant analysis algorithm, following the methods outlined in ISO 13321:1996E and ISO 22412:2008 [57,58,59]. The refractive index and viscosity of the water were used as calculation parameters for the dispersant and polystyrene latex materials. The HD and PDI data figures display the average values from five consecutive measurements at each temperature, with good agreement between repeated results. Zetasizer^®^ software version 7.10 was used to design custom standard operating protocols (SOPs). The SOPs were used to process data from the DLS measurements on subsequent samples without any modifications.

### 2.7. Zeta Potential (ZP) Measurements

The Zetasizer Nano ZS ZEN3600 device (Malvern Instruments, Malvern, UK) was used to measure the zeta potential (ZP) based on the laser Doppler electrophoresis technique (laser Doppler velocimetry, LDV) with the Zetasizer^®^ software (version 7.11). The electrophoretic mobilities (EM) of the polymer particles in the aqueous dispersion were measured and converted to ZP using the Smoluchowski model approximation to Henry’s equation (f(Ka) = 1.5). The DTS-1070 U-shaped capillary cuvette, made of polycarbonate plastic, with a capacity of 0.75 mL and equipped with a gold-plated copper electrode, (Malvern Instruments, Malvern, UK), was used to record the zeta potential. Measurements were taken at one-degree intervals between 18 and 45 °C, with a 120 **s** equilibration time for each temperature. The zeta potential values were calculated as the average of five measurements at each temperature.

## 3. Results

### 3.1. Synthesis

**Figure 2:**
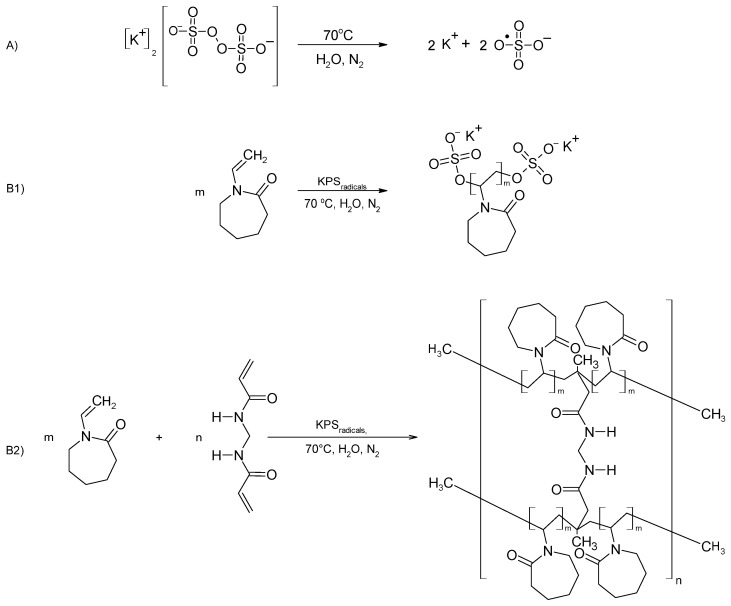
The general scheme proposed for NVCL polymerization: (**A**) generating radicals; (**B1**) polymerizing without a cross-linking agent; and (**B2**) polymerizing with the MBA cross-linking agent under the experimental conditions used in this study.

Six unitary syntheses were conducted to produce polymeric NVCL derivatives, named P1-P6, with varying starting compositions. Section 2.2 provides a detailed description of the polymerization reaction. The proposed polymerization reaction schemes are shown in Figure 2. A cross-linking agent was added in the synthesis of P5 and P6.

**Figure 3:**
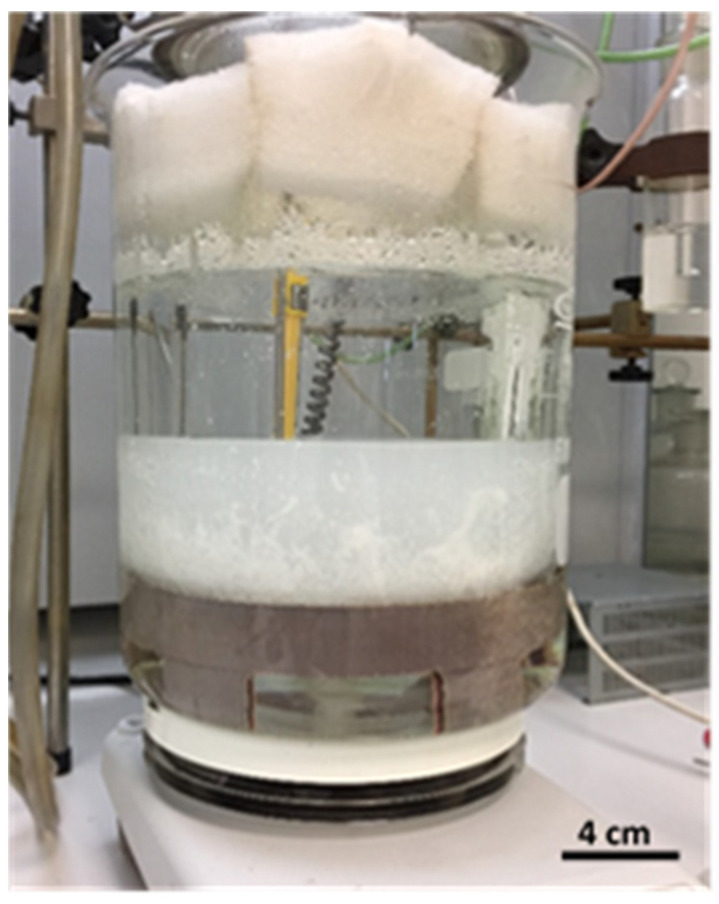
Turbidity was observed during the synthesis of polymer P5. The scale bar is 4 cm.

**Figure 4:**
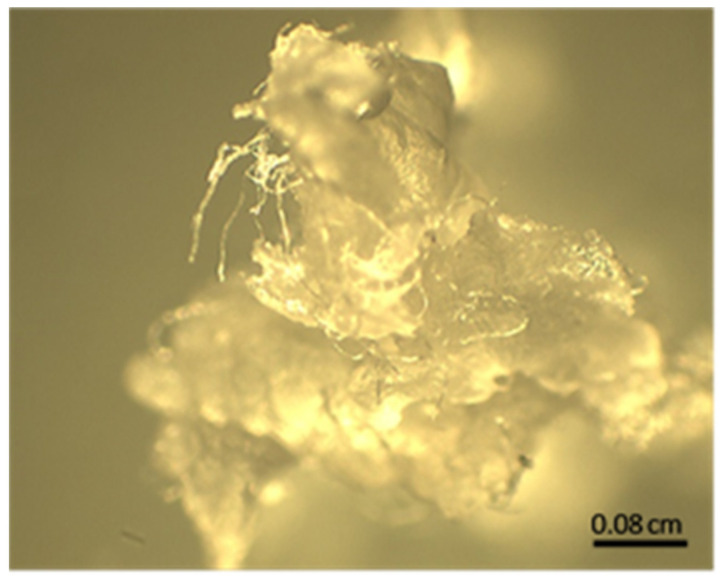
Optical microscopic image of a precipitate obtained during the synthesis of polymer P5. The scale bar is 0.08 cm.

Turbidity and precipitate formation occurred only in the synthesis of polymer P5, as shown in Figure 3. The collected precipitate was lyophilized and microscopically analyzed. Figure 4 shows microscopic images of the precipitate of polymer P5 taken with an optical microscope (×50).

### 3.2. Conductivity Measurements

**Figure 5:**
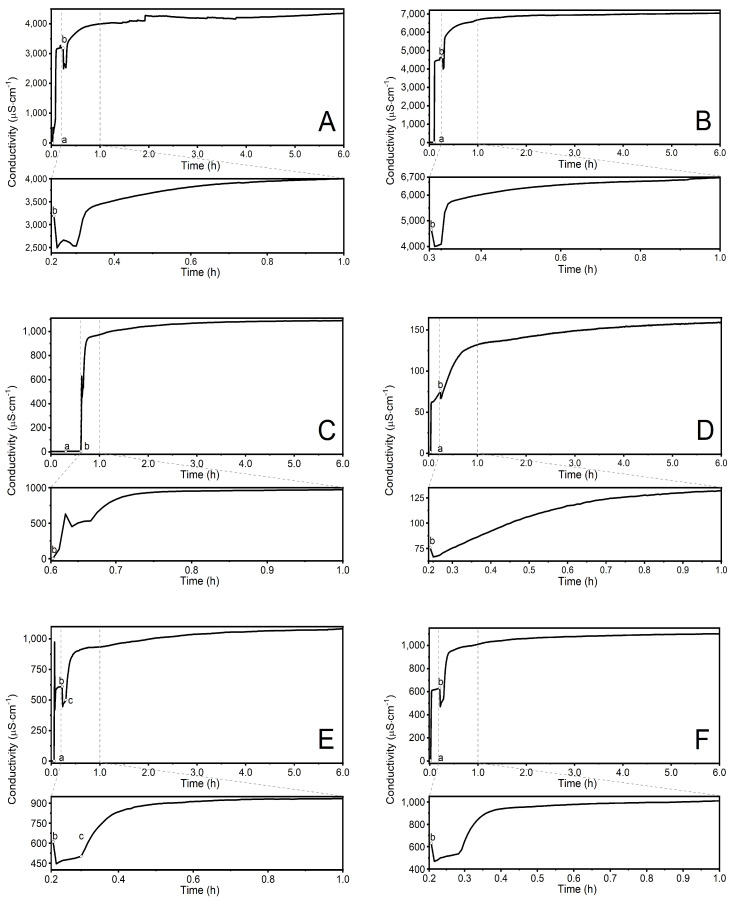
The changes in conductivity recorded over time in reaction systems P1 (**A**), P2 (**B**), P3 (**C**), P4 (**D**), P5 (**E**) and P6 (**F**) during synthesis at T = 70 °C. Point (a) marks the addition of the initiator KPS, point (b) marks the addition of an aqueous solution of monomer NVCL (Figure 4 and Figure 5A–D) or a mixture of monomer NVCL and cross-linker AMB (Figure 4 and Figure 5E,F), and point (c) marks the onset of visible change in the turbidity of the reaction mixture.

Changes in the electrolytic conductivity of the reaction system were continuously recorded to observe the progress of the reaction, as depicted in Figure 5A–F. No turbidity was observed during the polymerization of P1-P3 and P6 polymers in the reaction system. However, turbidity appeared in the reaction system during the polymerization of P5. This occurred approximately 200 s after the addition of the mixture of initiator and cross-linked agents, as illustrated in Figure 5E, point (c).

**Figure 6:**
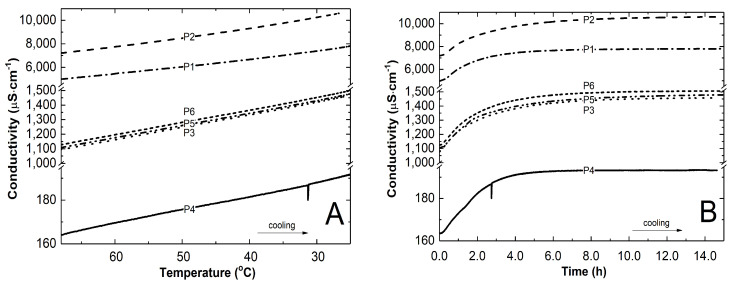
The changes in conductivity in P1–P6 post-reaction mixtures during the cooling procedure as a function of temperature (**A**) and time (**B**).

After six hours of polymerization, the heating was turned off. The reaction mixture was left to cool down to an ambient temperature for approximately 16 h. Figure 6 shows the variations in conductivity of the P1-P6 reaction mixtures in relation to temperature (Figure 6A) and time (Figure 6B) during the cooling of the polymer systems after polymerization. In all cases, the decrease in temperature led to an increase in conductivity.

### 3.3. pH Measurements

Table 2 shows the pH values of the reaction mixtures measured after cooling to room temperature, as well as the pH values of the reaction mixtures purified by forced equilibration dialysis (FED).

### 3.4. Attenuated Total Reflection Fourier Transform Infrared Spectroscopy Analysis (ATR-FTIR)

**Figure 7:**
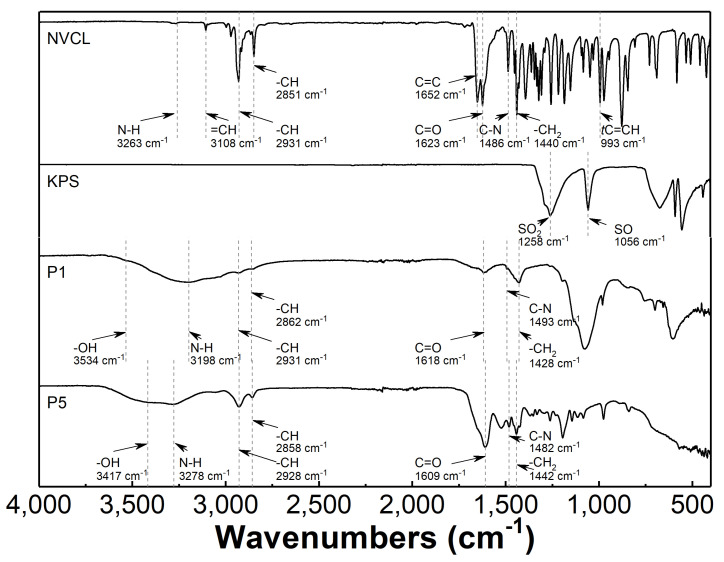
ATR-FTIR spectra of monomer—N-vinylocaprolactam (NVCL); initiator—potassium persulfate (KPS); synthesized polymers P1 and P5.

Infrared spectroscopy studies were carried out on the lyophilized polymer P1, the precipitate formed during the synthesis of polymer P5, as well as the commercial monomer NVCL and the initiator KPS which were the substrates in each reaction. Figure 7 shows the FTIR spectra with highlighted characteristic peaks. Table 3 compares the band positions recorded for the polymerization products P1 and P5 polymers and their corresponding functional groups with data available in the literature.

### 3.5. Hydrodynamic Diameter (HD) and Polydispersity Index (PDI) Measurements

**Figure 8:**
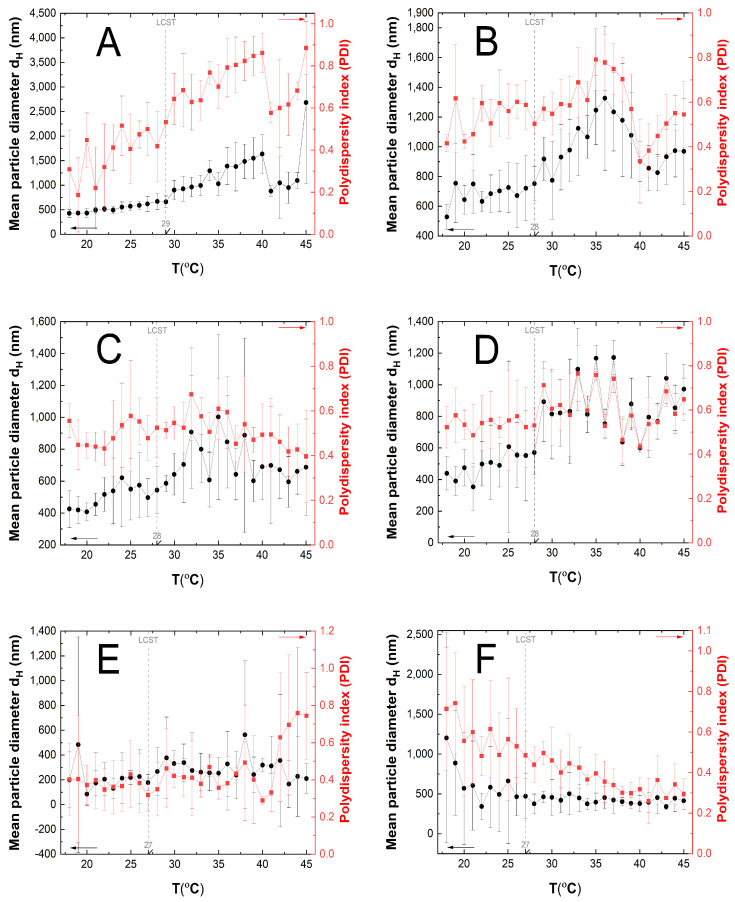
The hydrodynamic diameter (HD) and polydispersity index (PDI) changes with temperature increase and lower critical solution temperature (LCST) determined for polymeric particles P1 (**A**), P2 (**B**), P3 (**C**), P4 (**D**), P5 (**E**), and P6 (**F**), using the dynamic light scattering method.

Figure 8A–E displays the impact of temperature on the hydrodynamic diameters (HD) and polydispersity index (PDI) of particles in aqueous suspensions of polymers P1 (A), P2 (B), P3 (C), P4 (D), P5 (E), and P6 (F) within the range of 18–45 °C. Measurements were taken for samples that were purified by dialysis and were undiluted.

### 3.6. Zeta Potential (ZP) Measurements

**Figure 9:**
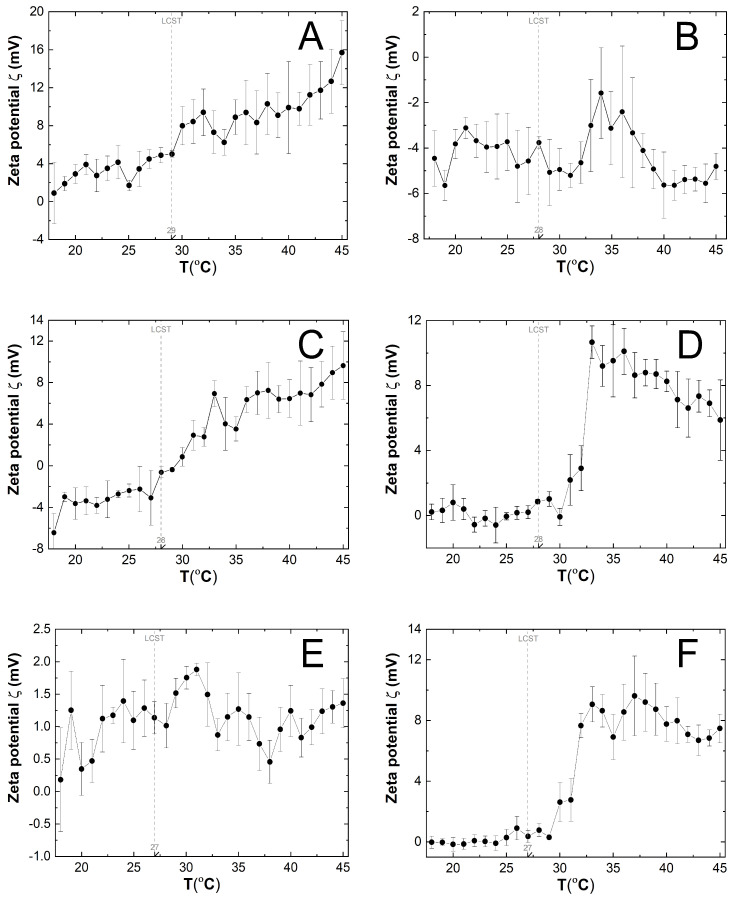
The zeta potential (ZP) changes with temperature increase, determined for polymeric particles P1 (**A**), P2 (**B**), P3 (**C**), P4 (**D**), P5 (**E**), and P6 (**F**), using the electrophoretic mobility method. The lower critical solution temperature (LCST) was determined by the dynamic light scattering (DLS) method.

Figure 9A–F illustrates the variations in zeta potential (ZP) of the P1-P6 purified polymer dispersions as a function of temperature, ranging from 18 to 45 °C. The measured samples were not buffered. The pH values of samples P1, P2, P3, P4, P5, and P6 were 5.29, 6.08, 5.00, 5.10, 4.80, and 4.85, respectively, at a temperature of 22 °C. The surface of polymer particles P1, P4, P5, and P6 had a positive zeta potential (ZP) between 18 and 45 °C. In contrast, polymer particles P2 had a negative charge throughout the entire temperature range. Polymer particles P3 had negative ZP values between 18 and 28 °C and positive values above 28 °C. The graphs P1, P2, P3, P4, and P6 show two areas of ZP change, defined by the temperature ranges before and after the phase transition. The ZP values of P5 polymers remained stable with minor deviations over the temperature range used.

## 4. Discussion

Six syntheses of *N-vinylcaprolactam* derivatives were conducted to examine the impact of initial factors on the physicochemical properties of the products and determine the most suitable product to serve as a carrier for medicinal substances.

### 4.1. Synthesis

Thermosensitive polymers were synthesized by polymerizing P1-P4 and P6 under the adopted synthesis conditions. The polymerization process was carried out at a temperature above the polymer phase transition temperature, based on the hydrophobicity of the increasing polymer chains. No turbidity of the phase transition was observed during the synthesis of the polymers P1-P4 and P6. Even at temperatures above the phase transition temperature, the intensity of light scattering from suspended particles remained low. The entire synthesis process can be significantly impacted by the reactivity and hydrolysis propensity of the initiator used in the solution. The ionization-capable terminal functional groups in the polymer chain can affect electrostatic interactions between the chains, leading to their soluble or coiled polymer form. The chains are suspended in a continuous medium as a polymer gel due to repulsive forces and enter the solution. It is also possible for the initiator to undergo several side reactions before the entering of the monomer solution into the reaction system. These side reactions may decrease the efficiency of homolytic cleavage, which generates the radicals initiating the polymerization process. At the low pH found in the reaction system under study, the reaction competing with the polymerization reaction may also occur, such as NVCL hydrolysis, resulting in a reduction of the available amount of monomer [37]. Moreover, during the nucleation stage, the chain growth may be deactivated more rapidly, resulting in the formation of oligomers that do not reach the critical chain length and therefore do not precipitate as precursor particles. The scattering intensity increases when P5 polymerizes, resulting in a milky white dispersion that is visible to the naked eye. The visibility of the dispersion may be a result of the addition of a cationic cross-linking agent at a weight ratio 1:1 to the initiator.

### 4.2. Conductivity

The conductivity data of the synthesis process show that the level of conductivity in the reaction mixture is influenced by the type and amount of substrate used, cf. Figure 5A–F. Comparing two syntheses, P1 and P2 (Figure 5A,B), it is evident the system’s conductivity increases with the same qualitative composition, but with relatively higher amounts of initiator and monomer. However, the profile of conductivity variation remains unchanged.

A decrease in the amount of initiator in the system led to a decrease in conductivity. Additionally, introducing a cross-linking agent into the reaction system resulted in a milder alteration in the conductivity profile, compared to systems without the cross-linking agent. Changes in the amount of initiator or the addition of a cross-linker were reflected in the conductivity change profiles over a short time interval of 300 s after introducing the monomer or monomer/cross-linker mixture into the system, as shown in the zoomed graphs at the bottom of Figure 5A–F. The variability of the conductivity profiles suggests a potential variation in the formation of polymer chains during the initial stages of the polymerization reaction. The conductivity results, obtained during the synthesis, confirm that the polymerization process and its end point can be determined using this electrochemical method.

By analyzing the results of the conductivity measurements of the post-reaction mixtures as they cooled, Figure 6A,B, the times and corresponding temperatures at which the conductivity stabilized at a constant level were determined. The temperatures at which the conductivity acquired plateau for P1-P6, decoded from comparisons of Figure 6A,B, were 25.9 °C after 27,150 s, 29.0 °C after 34,500 s, 26.6 °C after 37,500 s, 23.8 °C after 20,820 s, 26.8 °C after 37,470 s, and 25.4 °C after 30,630 s. It was expected that conductivity values will reflect phase transition temperatures, according to former experiments [61], but this was not the case. The phase transition temperature appears to be more influenced by the presence of unreacted initiator and monomer particles, as well as other possible intermediates, in the untreated reaction system, whose pH is lower than that of the purified system, than previously anticipated.

### 4.3. pH

Table 2 presents the results indicating that the pH values of the untreated polymer solutions were acidic, ranging from 1.67 to 3.50. The purification process reduced the acidity, resulting in higher pH values between 4.80 and 6.08 for the purified polymer solutions. These values are similar to the physiological pH values of human skin [62,63,64], indicating that the synthesized products are compatible with the acidity of superficial layers of human dermis [65].

### 4.4. ATR-FTIR

Infrared spectroscopy studies confirmed that the polymerization reaction occurs by the opening of the vinyl bond. Figure 7 and Table 3 provide data to support this conclusion. The ATR-FTIR spectra of the monomer and polymers P1 and P5 show overlapping absorption bands due to vibrations in the N-H, C-H, C=O, C-N, and -CH2- functional groups. The position of these bands in the compared spectra is comparable. The FTIR spectrum of the polymers does not show absorption bands originating from double bonds in the C=C and =CH2 functional groups resulting from the polymerization reaction. Instead, an absorption band indicating the presence of an O-H bond is observed, which may be due to the presence of water in the polymer chains [60,66,67].

### 4.5. HD and PDI

**Figure 10:**
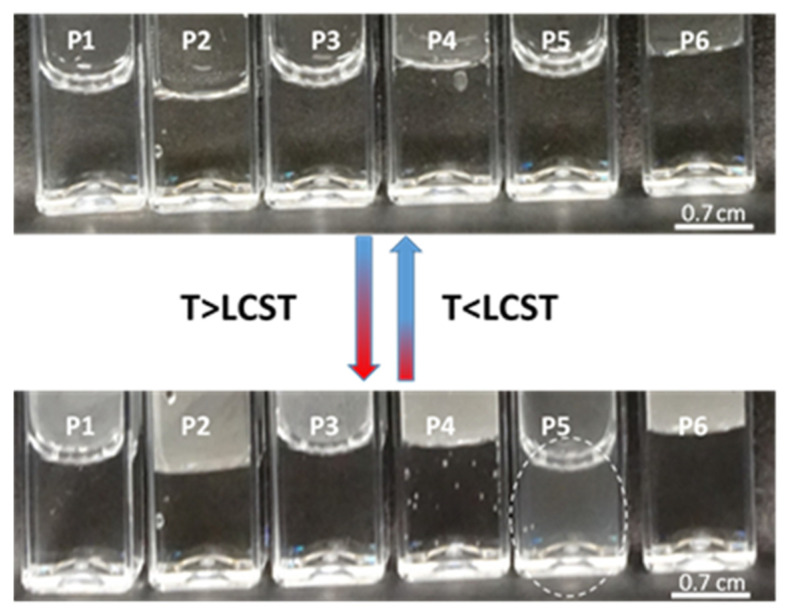
Photos of purified samples of P1-P6 polymers at room temperature ~25 °C (**top**) and heated to 45 °C (**bottom**). The scale bar is 0.7 cm.

The size of the synthesized polymers was determined by measuring their hydrodynamic diameter (HD) to assess their suitability as carriers for therapeutic particles for systemic application. A temperature-dependent test was carried out to confirm their thermosensitivity. According to the macroscopic images of the macromolecules, only in the case of P5 was the phase transition visualized, as indicated by the dotted line circle in Figure 10. However, the HD measurements enabled the further evaluation of the dispersions of the polymers (Figure 10).

The hydrodynamic diameter of all polymers changed with increasing temperature, but the changes were less pronounced for both uncross-linked and cross-linked polymers, as compared to data reported in the literature [65,68]. The hydrodynamic diameter values underwent significant changes between 27 and 29 °C, indicating a phase transition of the polymer in this temperature range, convergent with the literature data [38,39,40]. Polymer P1 exhibited the highest phase transition temperature, while polymers P5 and P6 differed from the others by approximately one degree. These results suggest that, under the given experimental conditions, cross-linking did not have a significant effect on the phase transition temperature of the tested systems.

The polydispersity indexes were measured to evaluate the homogeneity of the polymer particles [69,70]. The polydispersity index is a numerical value that ranges from 0.0 to 1.0. Lower PDI values indicate more uniform or monodisperse samples. The results showed differentiation among the particles as the temperature increased, which is consistent with the hydrodynamic diameter measurements with high accuracy. The PDI coefficients indicate a relatively low polydispersity of P3 and P4 polymers. An increase in polydispersity above the estimated phase transition temperatures may be related to particle aggregation [71]. The PDI value was not exceeded above unity in any measured system.

### 4.6. ZP

The zeta potential significantly influences the interaction of particles with their environment; it can also determine the long-term stability of colloidal systems and can be used to study surface properties and related adsorption phenomena [72,73]. The zeta potential measurements conducted in this study indicated a significant change in ZP values above temperatures corresponding to the phase transition temperatures estimated using the DLS method. However, only a moderate change was observed in polymer P3. Additionally, the zeta potential values for P3 decreased above the phase transition temperature. At 25 °C, zeta potential measurements indicated a negative charge on the particle surface of polymers P2 and P3 as well as P6. The zeta potential values for P1, P4, P5, and P6 polymers were close to zero. The increase in zeta potential above the LCST temperature for polymers P1 and P3 was related to polymer aggregation rather than improved stability at higher temperatures. Polymers P2, P4, and P6 also exhibited an increase in zeta potential above the LCST temperature, but this increase was not monotonic and remained constant up to 45 °C. The system was considered to be unstable when the assumed zeta potential value was between −30 and 30 mV. The polymers dispersions that were tested exhibited a zeta potential ranging from −6.44 to 15.7 mV across the temperature range.

## 5. Conclusions

This study investigated the influence of initial reaction factors on the physicochemical parameters of the obtained products. The polymerization process, under the given conditions, resulted in the formation of P1-P6 polymers that exhibit reversible sensitivity to temperature, as evidenced by a change in particle size within the temperature range of 27–29 °C. The phase transition temperature was not significantly impacted by variations in initiator concentration. However, when considering the influence of external factors on LCST, it is crucial to take into account electrostatic interactions, hydrophobic forces, and ion pair formation. Therefore, in future studies, polymerization should be carried out by altering the pH values, using a cationic initiator, or applying a buffer. The polymer particles have hydrodynamic diameters ranging from 204 to 632 nm at 22 °C. The study demonstrated that the addition of a cross-linking agent significantly reduced the hydrodynamic diameter of the polymer particles. Zeta potential measurements confirmed that the synthesized polymers tend to aggregate. In summary, preliminary pH and HD tests confirmed their potential and validity as materials for the development of drug carriers for skin applications. In addition, conductivity measurements can be used to determine the finalization of the radical polymerization reaction in polymer synthesis and to visualize the course of the polymerization reaction.

## Figures and Tables

**Table 1:** Substrate compositions of P1, P2, P3, P4, P5, and P6 thermosensitive polymers.

Components		Polymer Code					
		P1	P2	P3	P4	P5	P6
Monomer (g)	NVCL	3.00	5.00	5.00	5.00	5.00	5.00
Anionic initiator (g)	KPS	2.91	4.85	0.49	0.049	0.49	0.49
Cross-linker (g)	MBA		-	-	-	0.55	0.0055

**Table 2:** The pH values of unpurified and purified synthesis products.

Polymer Code	Polymer Dyspersion	
	Unpurified before FED	Purified after FED
P1	1.90	5.29
P2	1.67	6.08
P3	2.45	5.00
P4	3.50	5.10
P5	2.50	4.80
P6	2.50	4.85

**Table 3:** Characteristic FTIR absorption bands of the NVCL monomer, P1, and P5 polymers.

Assignation	Wavenumbers $\tilde{v}$/cm^−1^			
	Reference [60]	Observed		
	NVCL/ pNVCL	NVCL	P1	P5
N-H	3274	3263	3198	3278
C-H	2926 2856	2931 2851	2931 2862	2928 2858
C=O	1631	1623	1618	1609
C-N	1479	1486	1493	1482
-CH_2_-	1441	1440	1428	1442
C=C	1659	1652	-	-
=CH_2_	994	993	-	-
O-H	3507	-	3534	3417

## Data Availability

The original contributions presented in the study are included in the article, further inquiries can be directed to the corresponding author.
